# Physicochemical and sensory attributes assessment of functional low‐fat yogurt produced by incorporation of barley bran and *Lactobacillus acidophilus*

**DOI:** 10.1002/fsn3.470

**Published:** 2017-04-24

**Authors:** Saber Hasani, Abbas Ali Sari, Ali Heshmati, Mostafa Karami

**Affiliations:** ^1^ Department of Food Hygiene and Quality Control Faculty of Veterinary Science Bu‐Ali Sina University Hamadan Iran; ^2^ Nutrition Health Research Center Hamadan University of Medical Sciences Hamadan Iran; ^3^ Department of Food Science and Technology College of Food Science and Technology of Bahar Bu‐Ali Sina University Hamadan Iran

**Keywords:** barley bran, *Lactobacillus acidophilus*, low‐fat yogurt

## Abstract

The aim of this study was to analyze the effect of different amounts of barley bran (0.3%, 0.6%, 0.9%, and 1.2%) on the viability of *Lactobacillus acidophilus* and the physicochemical and sensory properties of low‐fat yogurt during storage period (28 days). Results showed that *L. acidophilus* number and viscosity in samples containing barley bran was significantly higher than the control group (*p *<* *.05). High levels of barley bran (1.2%) decreased sensory prosperity scores and led to viscosity increment; although sensory prosperity scores of samples containing 0.6% barley bran did not show significant difference with control sample, while the number of *L. acidophilus* in this treatment was higher than minimal acceptable level (10^6^ CFU/g). Therefore, level of 0.6% of barley bran is recommended for symbiotic yogurt production. According to the present study, a positive correlation was observed between barley bran concentrations in the yogurt with *L. acidophilus* number.

## 1 INTRODUCTION

In recent years, the nutritional value and health‐promoting properties of foods are the main concerns of consumers. In this regard, symbiotic foods that have both probiotic and prebiotic properties were considered (Krasaekoopt, Bhandari, & Deeth, 2004). Probiotics are live microbial food products and supplements with beneficial health effects on the consumers by maintaining or improving intestinal microbiota (Nagpal et al., 2012). Prebiotics are nondigestible compounds that selectively stimulate the growth of probiotic bacteria and impart health benefits to the consumer (Gorinstein et al., 2001). Cereal fibers are the main types of prebiotics. The residual compounds from cereal process are important sources for fibers. In food production, the fibers are being used as filler and low‐cost ingredients (Sendra et al., 2008). Insufficient intake of fibers in food regimes is one of the main nutritional concerns that causes different problems and gastrointestinal diseases. Therefore, enrichment of foods with cereal fibers is a good choice for improving daily intake of fibers (Davidson & Mcdonald, 1998). Daily intake of 38 and 25 g of fiber has been recommended for men and women, respectively (Sendra et al., 2008). The significant healthy effects of cereal bran on the reduction of blood sugar, cholesterol level, and risk of intestinal diseases have been proved. Barley bran due to its polysaccharide, hemicellulose (Thiago & Kellaway, 1982), and water soluble and insoluble fibers (β‐glucan and cellulose, respectively) exhibit beneficial health effects (Davidson & Mcdonald, 1998). According to FAO/WHO recommendation, more than 10^7^ CFU/g of probiotic bacteria in yogurt at the time of consumption is necessary. Usually due to high acid content, presence of bacteriocins, and fermentation conditions, the survival ability of probiotic bacteria decreased before consumption Ferdousi et al., 2013). Therefore, utilization of prebiotic compounds is a common method in order to increase the survival ability of these bacteria at the consumption time (Pharmaceutiques, 1995). Cereal fibers can be selectively metabolized by intestinal microbiota and increase the probiotic bacterial numbers (Sendra et al., 2008). In recent years, due to important role of probiotic and dietary fibers on human health, a great attention has been paid on the application of dietary fibers in the production of different dairy products, especially yogurt.

In a study, with incorporation of wheat bran in fruit yogurt, the highest viscosity and lowest syneresis were found in yogurt with 0.6% wheat bran. Sendra et al. (2008) reported that incorporation of citrus fiber into fermented milk leads to increased growth and viability of probiotics (Sendra et al., 2008). The study of Capela, Hay, and Shah (2006) showed that 1.5% inulin in yogurt formulation increased viability of *Lactobacillus acidophilus* and *L. casei* (1.42 log_10_ CFU/g) during storage at 4°C for 4 weeks (Capela et al., 2006). Also, addition of 5% inulin to cottage cheese increased number of *L. delbrueckii* during storage period (Capela et al., 2006). The addition of the date fiber and wheat bran in yogurt showed that yogurt containing 3% date fiber has a different color compare to control treatment, but performed a good consumer acceptance (Hashim, Khalil, & Afifi, 2009). The aim of this study was to investigate the effects of barley bran on the growth of *L. acidophilus* and other quality attributes such as sensory and physicochemical properties in low‐fat yogurt.

## 2 MATERIALS AND METHODS

### 2.1 Materials

Low‐fat milk (1%) and commercial starter YC‐X1 containing *Streptococcus thermophilus* and *L. delbrueckii* subsp. *bulgaricus* were obtained from Pegah, Co. (Hamadan, Iran). The barley bran was purchased from the Sina flour company (Hamadan, Iran) and was used after grinding and sieving using 70″ mesh screen. Other chemicals were purchased from Merck (Darmstadt, Germany).

### 2.2 Preparation of *L. acidophilus* ATCC 4356

Lyophilized cultures of *L. acidophilus* ATCC 4356 obtained from the Department of Microbiology, Faculty of Veterinary Medicine, University of Tehran, Iran, were used in this study. The lyophilized cultures were grown twice in tubes containing 10 ml of MRS broth (Quelab, Co., Canada) at 37°C for 18 hr. Then, bacterial suspension with 2 McFarland turbidity (6 × 10^8^ CFU/ml) was prepared from the second culture (Krasaekoopt et al., 2004).

### 2.3 Physicochemical assessment and microbial quantification of barley bran

The moisture content, ash, acidity, and pH value of barley bran were analyzed. For total microbial count, at first, 10‐fold serial dilution of barley bran was prepared and then 0.1 ml of each dilution was plated on nutrient agar by surface plating method. All plates were incubated at 37°C for 24–48 hr. Plates containing 30–300 colonies were selected, and the number of colonies were counted and reported as total microbial count (CFU/g). Also, mold and yeast count were done on plates containing potato dextrose agar. Inoculated plates were incubated at 25°C for 3–5 days, and plates containing 15–150 colonies were counted (AOAC, 2005).

### 2.4 Preparation of yogurt

After standardization of solid contents and adding different amounts of barley bran (0.3%, 0.6%, 0.9%, and 1.2% w/v), raw milk was treated at 95°C for 5 min and cooled to 42°C. Then, 2% of fresh commercial starter culture (YC‐X11) along with 1% of adjusted *L. acidophilus* suspension with 2 McFarland turbidity were added to yogurt. All treatments were incubated at 42°C for 4 hr and then stored at 4°C for 28 days (Moreira, Abraham, & De Antoni, 2000). Number of *L. acidophilus* bacteria, viscosity, acidity, and pH of all treatments were determined at 1, 7, 14, 21, and 28 days. Sensory evaluation was done on the seventh day.

### 2.5 Measurement of pH and acidity

The pH of yogurt samples was measured at the time interval of 0, 7, 14, 21, and 28 days of storage periods by using the pH meter (Thermo Orion pH meter, model 420 Waltham, MA). The titratable acidity of yogurt samples was measured by the titration method. For this purpose, 10 g of yogurt were mixed with 20 ml of sterile distilled water and then this slurry solution was titrated with 0.1 N NaOH. Phenolphthalein was used as the indicator and acidity was expressed as the lactic acid percent (AOAC, 2005).

### 2.6 Quantification of *L. acidophilus*

Twenty‐five grams of yogurt sample was mixed with 225 ml of 0.1% sterile peptone water. Tenfold serial dilutions were prepared by adding 1 ml of each dilution to 9 ml of sterile peptone water. Then, 0.1 ml of each dilution was inoculated in MRS‐Bile agar (containing 0.15% bile salt) during surface plating method, and after incubation at 37°C for 72 hr, number of colonies on selected plates were counted (Karimi, Mortazavian, & Amiri‐Rigi, 2012).

### 2.7 Sensory evaluation

According to the method of Institute of Standards and Industrial Research of Iran, a 5‐point facial hedonic scale was used for sensory evaluation of yogurt. At first, each treatment was encoded randomly and evaluation procedure was performed. Flavor, mouth texture, nonmouth texture (yogurt behavior during stirring with spoon), and appearance attributes of each treatment were evaluated by 15 trained panelists. The score of each sensory attribute was multiplied by 6, 3, 2, and 1, respectively.

### 2.8 Viscosity measurement

The apparent viscosity of samples was measured using a rotary Brookfield Viscometer (RVDV2, Brookfield, MA, USA) with RV4 spindle. Before starting the test, all samples were kept at 7°C in constant conditions to remove any stress or change in their texture. Viscosity of each treatment was carried out at 80 rpm shear stress during 60 s. The test type was as single point (Trachoo & Mistry, 1998).

### 2.9 Chemical analysis of raw milk

Fat, solid nonfat, protein, acidity, and pH value of raw milk were 1.1%, 8.2%, 3.15%, 0.15%, and 6.7, respectively.

### 2.10 Chemical and microbial analysis of barley bran

Moisture and ash contents, acidity, pH, and mold and microbial counts of barley bran were 7%, 6.2%, 0.45%, 6.4, 1.5 × 10^2^ ****CFU/g, and 3 × 10^2^ CFU/g, respectively.

### 2.11 Statistical analysis

All experiments were replicated three times. Data analysis was done using SPSS software, version 16.0 (Chicago, IL, USA). Mean comparison were determined by Tukey's test (*p *<* *.05). All data were reported as the mean ± standard deviation (*SD*).

## 3 RESULTS AND DISCUSSION

### 3.1 Growth response of *L. acidophilus*

The effect of different concentrations of barley bran on *L. acidophilus* viability in stirred yogurt is shown in Table 1. Results showed that barley bran concentration and storage time had a significant effect on bacterial growth (*p *<* *.05). On the first day and after incubation, the amount of *L. acidophilus* in treatments containing barley bran was significantly higher than control group; the number of *L. acidophilus* bacteria was 7.7 log CFU/g and 7.2 log CFU/g in treatments containing 1.2% of barley bran and control group, respectively. In general, increase in barely bran concentration increased the bacterial counts, as the highest number of *L. acidophilus* was observed in treatments containing 1.2% of barley bran. Also, in each treatment losses number of *L. acidophilus* were lower than control group, as 0.57 and 0.36 log CFU/g lose of *L. acidophilus* was observed at 28 days of storage in control group and yogurt containing 1.2% of barley bran, respectively. Generally, in presence of barley bran, survivability of *L. acidophilus* in low‐fat yogurt was significantly increased compared to the control treatment. This may be due to presence of starchy and nitrogenous components and the presence of structural polysaccharides such as β‐glucan in barley bran (Desai, Powell, & Shah, 2004; Makras, Va Nacker, & De Vuyst, 2005). Results showed that the number of *L. acidophilus* in all treatments containing barley bran at 28 days of storage time was higher than the minimum recommended probiotic number for exhibiting treatment effect (10^7^ CFU/g). According to our results, barley bran showed prebiotic effect and improved the growth of *L. acidophilus* in yogurt. These findings were consistent with other previous studies. Saarela, Virkajärvi, Nohynek, Vaari, and Mättö (2006) showed that barley bran has a higher effect on viability of *L. casei* than inulin and apple fiber in apple juice and chocolate‐coated breakfast cereals (Saarela et al., 2006). In their study, Zomorodi, Aberoon, and Khosrowshahi (2015) observed that wheat bran and apple fiber increased survivability of *L. acidophilus* in yogurt as the number of *L. acidophilus* in treatments containing 1% wheat bran increased 0.25 log CFU/g, whereas in control group, number of *L. acidophilus* decreased 1 log CFU/g (Zomorodi et al., 2015). Akin, Akin, and Kirmaci (2007) observed that incorporation of inulin and sugar in probiotic yogurt and ice cream increased the number of *L. acidophilus* and *Bifidobacterium lactis* (Akin et al., 2007). Capela et al. (2006) showed that 1.5% of raftilose in yogurt increased the viability of some probiotic bacteria including *L. acidophilus*,* L. casei*,* L. rhamnosus*, and *Bifidobacterium* during 4 weeks of storage at 4°C (Capela et al., 2006). In study of Lourens‐Hattingh and Viljoen (2001), survivability of *L. acidophilus* at day 28 compared to first day was higher than the control group.

**Table 1:** The *Lactobacillus acidophilus* number (log_10 _CFU/ml) of different yogurt formulations during storage period (mean ± *SD*)

Yogurt formulation	Day				
	0	7	14	21	28
T1	7.77 ± 0.26^a^	7.63 ± 0.14^a^	7.55 ± 0.14^a^	7.47 ± 0.14^a^	7.41 ± 0.02^a^
T2	7.61 ± 0.21^ab^	7.51 ± 0.08^ab^	7.44 ± 0.09^ab^	7.33 ± 0.13^ab^	7.21 ± 0.13^b^
T3	7.52 ± 0.38^b^	7.45 ± 0.06^b^	7.34 ± 0.06^b^	7.27 ± 0.15^b^	7.13 ± 0.12^bc^
T4	7.42 ± 0.07^bc^	7.34 ± 0.19^bc^	7.25 ± 0.06^bc^	7.11 ± 0.06^c^	7.06 ± 0.01^c^
B1	7.22 ± 0.05^c^	7.17 ± 0.16^c^	7.05 ± 0.57^c^	6.91 ± 0.13^c^	6.63 ± 0.36^d^

T1, T2, T3, T4: yogurt containing 1.2%, 0.9%, 0.6%, 0.3% concentration of barley bran, respectively. B1: yogurt containing probiotic bacteria without barley bran.

### 3.2 Physicochemical properties

#### 3.2.1 The pH and acidity

Results showed that the barley bran concentration and presence of *L. acidophilus* has a significant effect on the pH value and acidity of treatment groups (*p *<* *.05). The pH value of all treatments during the storage period at 4°C was significantly lower than control group. The acidity value of all samples containing barley bran and *L. acidophilus* was significantly higher than control groups. In accordance with our results, Fernandez‐Garcia and Mcgregor (1997) reported that 1.3% of barley fiber leads to increase in acidity and decrease in pH of yogurt.

#### 3.2.2 Viscosity

The effect of barley bran on the viscosity of different treatments has been indicated in Table 2. As can be seen, treatments containing 1.2% of barley bran at the first day had the highest viscosity. This phenomenon is correlated with the amount of barley bran, while the lowest viscosity was observed in control group. During the storage period, the viscosity of samples containing barley bran decreased with an irregular pattern. It may be due to the barley bran sedimentation that does not allow the proteins to rearrange and so the viscosity of these treatments decreases, whereas in control group (without barley bran), the viscosity increased with a regular pattern, it will be due to the rearrangement of yogurt gel proteins and retaining of water by hydrophobic proteins (Staffolo, Bertola, Martino, & Bevilacqua, 2004). Among all treatments, except to the control group, at day 28, the highest and lowest viscosities were related to the treatments containing 1.2% and 0.6% of barley bran, respectively (*p *<* *.05). This may be due to breaking effect of barley bran into the gel network (Fernandez‐Garcia & Mcgregor, 1997). While the barley bran breaks the gel network, but in higher concentrations leads to increase in the viscosity of the final product that may be due to the high level of water absorption (Elleuch et al., 2011). Another researcher indicated that corn, rice, and barley fiber increase the final product viscosity due to the interaction between milk proteins and oligosaccharides and polysaccharides of barley bran (Fernandez‐Garcia & Mcgregor, 1997). Garcia‐Perez et al. (2006) indicated that orange fiber due to water holding capacity improves the rheological properties of yogurt because the water is absorbed by the fiber.

**Table 2:** Variation in the pH and acidity values of different yogurt formulations during storage period (mean ± *SD*)

Yogurt formulation	Day									
	0		7		14		21		28	
	Acidity	pH	Acidity	pH	Acidity	pH	Acidity	pH	Acidity	pH
T1	0.92 ± 0.03^a^	4.31 ± 0.11^b^	0.94 ± 0.02^a^	4.29 ± 0.06^c^	0.95 ± 0.03^a^	4.26 ± 0.1^bc^	0.97 ± 0.02^a^	4.23 ± 0.02^b^	0.99 ± 0.02^a^	4.18 ± 0.05^bc^
T2	0.88 ± 0.01^a^	4.37 ± 0.02^b^	0.88 ± 0.01^ab^	4.34 ± 0.07^bc^	0.9 ± 0.02^ab^	4.28 ± 0.01^bc^	0.90 ± 0.05^ab^	4.26 ± 0.01^ab^	0.92 ± 0.01^ab^	4.22 ± 0.06^b^
T3	0.86 ± 0.01^a^	4.4 ± 0.02^ab^	0.89 ± 0.06^ab^	4.33 ± 0.03^bc^	0.9 ± 0.02^ab^	4.3 ± 0.05^b^	0.90 ± 0.01^ab^	4.3 ± 0.40^ab^	0.93 ± 0.01^ab^	4.23 ± 0.1^b^
T4	0.82 ± 0.01^ab^	4.43 ± 0.11^ab^	0.86 ± 0.01^b^	4.39 ± 0.02^ab^	0.89 ± 0.02^ab^	4.35 ± 0.06^b^	0.89 ± 0.01^ab^	4.32 ± 0.01^ab^	0.89 ± 0.01^b^	4.26 ± 0.04^b^
B1	0.84 ± 0.02^ab^	4.41 ± 0.09^ab^	0.86 ± 0.2^b^	4.4 ± 0.02^ab^	0.88 ± 0.05^ab^	4.39 ± 0.14^ab^	0.89 ± 0.04^ab^	4.33 ± 0.11^ab^	0.9 ± 0.01^b^	4.29 ± 0.01^b^
B2	0.80 ± 0.01^b^	4.5 ± 0.04^a^	0.82 ± 0.07^b^	4.46 ± 0.03^a^	0.82 ± 0.05^b^	4.44 ± 0.01^a^	0.82 ± 0.05^b^	4.43 ± 0.02^a^	0.85 ± 0.02^c^	4.41 ± 0.02^a^

T1, T2, T3, T4: yogurt containing 1.2%, 0.9%, 0.6%, 0.3% concentration of barley bran, respectively. B1: yogurt containing probiotic bacteria without barley bran and B2 is the natural yogurt (containing nonprobiotic and fiber).

#### 3.2.3 Sensory evaluation

Determination of sensory properties is one of the most important methods to improve the product quality and evaluation of customer acceptance, as well as is a key parameter for differentiating between competitors and also affects the consumer attitude. According to the results, with increase in barley bran concentration, taste, appearance, and nonmouth texture scores decreased significantly (*p *<* *.05). The overall score of treatment containing 0.3% barley bran and control group was 40.4 and 41 from 50, respectively. Also, treatment containing 1.2% barley bran received the lowest score (*p *<* *.05). The similar results were reported by other researchers. Fernandez‐Garcia and Mcgregor (1997) reported that with the increase in the amounts of fibers, the consistency and texture of yogurt were improved, but its sensory quality decreased. Zomorodi showed that the sensory scores of samples were decreased by incorporation of wheat bran and apple fiber in probiotic yogurt, and the best sensory score was obtained in sample containing 0.5% wheat bran (Zomorodi et al., 2015). Hashim et al. (2009) reported that by incorporation of 4.5% date fiber in yogurt, the sensory scores of product decreased extensively; as vanillin was not able to reduce the unfavorable taste of the product. However, a good sensory score was obtained in yogurt containing 3% date fiber (Tables 3 and 4).

**Table 3:** Variation in the viscosity in different yogurt formulations during storage period (mean ± *SD*)

Yogurt formulation	Day				
	0	7	14	21	28
T1	1704 ± 82^a^	1386 ± 65^a^	1577 ± 3.7^a^	1441 ± 8.5^a^	1481 ± 29^a^
T2	1211 ± 56^b^	1146 ± 31^b^	1558 ± 1.5^a^	1153 ± 15^c^	1200 ± 10^c^
T3	724 ± 4.8^d^	830 ± 13^e^	751 ± 7.6^e^	777 ± 4.4^e^	784 ± 4.5^f^
T4	920 ± 5^c^	966 ± 13^cd^	969 ± 6.2^d^	860 ± 3.5^d^	864 ± 5^e^
B1	616 ± 15^d^	913 ± 14^ce^	1037 ± 26^c^	1128 ± 24^c^	1142 ± 15^d^
B2	644 ± 6.7^d^	1008 ± 27^c^	1156 ± 36^b^	1230 ± 12^b^	1254 ± 15^b^

T1, T2, T3, T4: yogurt containing 1.2%, 0.9%, 0.6%, 0.3% concentration of barley bran, respectively. B1: yogurt containing probiotic bacteria without barley bran and B2 is the natural yogurt (containing nonprobiotic and fiber).

**Table 4:** Sensory attributes evaluation of different yogurt formulations (mean ± *SD*)

Yogurt formulation	Taste	Mouth feel texture	Nonmouth feel texture	Appearance	Total score
T1	14.8 ± 3.8^b^	9.4 ± 3.2^a^	2.1 ± 0.83^d^	3.3 ± 1.4^d^	29.8 ± 8^c^
T2	16.4 ± 5.3^ab^	9.5 ± 2.7^a^	2.5 ± 0.91^bc^	4 ± 1.8^cd^	32.5 ± 9^bc^
T3	19.2 ± 3.3^ab^	10.2 ± 2.7^a^	3.1 ± 0.83^ab^	5.3 ± 1.7^bc^	37.93 ± 6.7^ab^
T4	19.6 ± 4.2^a^	10.9 ± 3.2^a^	3.4 ± 0.83^a^	6.4 ± 1.5^ab^	40.43 ± 8.1^ab^
B1	19.2 ± 4.6^ab^	10.9 ± 2.2^a^	3.4 ± 0.63^a^	7.06 ± 1.03^a^	40 ± 7.4^ab^
B2	19.6 ± 4.2^a^	11.2 ± 2.3^a^	3.4 ± 0.5^a^	6.8 ± 1.2^ab^	41 ± 6.6^a^

T1, T2, T3, T4: yogurt containing 1.2%, 0.9%, 0.6%, 0.3% concentration of barley bran, respectively. B1: yogurt containing probiotic bacteria without barley bran and B2 is the natural yogurt (containing nonprobiotic and fiber).

## 4 CONCLUSION

Incorporation of barley bran in low‐fat yogurt containing *L. acidophilus* significantly affected viable number of probiotic bacteria in comparison with control group. According to the present study, a positive correlation was observed between barley bran concentrations in the yogurt with *L. acidophilus* number. However, high levels of barley bran (1.2%) decreased sensory prosperity scores and led to viscosity increment, sensory prosperity scores of samples containing 0.6% barley bran did not show significant difference with control sample while the number of *L. acidophilus* in this treatment was higher than minimal acceptable level (10^6^ CFU/g). Therefore, level of 0.6% of barley bran is recommended for symbiotic yogurt production. However, it is recommended to use authorized additives and flavoring ingredients to improve the sensory properties of functional low‐fat yogurt containing *L. acidophilus* and high levels of barley bran.

## CONFLICT OF INTEREST

None declared.

## References

[fsn3470-bib-0001] Akin, M. B. , Akin, M. S. , & Kirmaci, Z. (2007). Effects of inulin and sugar levels on the viability of yogurt and probiotic bacteria and the physical and sensory characteristics in probiotic ice‐cream. Food Chemistry, 104, 93–99.

[fsn3470-bib-0200] AOAC . (2005). Official methods of analysis, 18th edition. Washington, DC: Association of Official Analytic Chemists.

[fsn3470-bib-0002] Capela, P. , Hay, T. , & Shah, N. (2006). Effect of cryoprotectants, prebiotics and microencapsulation on survival of probiotic organisms in yoghurt and freeze‐dried yoghurt. Food Research International, 39, 203–211.

[fsn3470-bib-0003] Davidson, M. H. , & Mcdonald, A. (1998). Fiber: Forms and functions. Nutrition Research, 18, 617–624.

[fsn3470-bib-0004] Desai, A. , Powell, I. , & Shah, N . (2004). Survival and activity of probiotic lactobacilli in skim milk containing prebiotics. Journal of Food Science, 69, FMS57–FMS60.

[fsn3470-bib-0005] Elleuch, M. , Bedigian, D. , Roiseux, O. , Besbes, S. , Blecker, C. , & Attia, H. (2011). Dietary fibre and fibre‐rich by‐products of food processing: characterisation, technological functionality and commercial applications: A review. Food Chemistry, 124, 411–421.

[fsn3470-bib-0006] Ferdousi, R. , Rouhi, M. , Mohammadi, R. , Mortazavian, A. M. , Khosravi‐Darani, K. , & Homayouni Rad, A. (2013). Evaluation of probiotic survivability in Yogurt exposed to cold chain interruption. Iranian Journal of Pharmaceutical Research, 12, 139–144. 24250681 PMC3813376

[fsn3470-bib-0007] Fernandez‐Garcia, E. , & Mcgregor, J. U. (1997). Fortification of sweetened plain yogurt with insoluble dietary fiber. Zeitschrift für Lebensmitteluntersuchung und‐Forschung A, 204, 433–437.

[fsn3470-bib-0008] Garcia‐Perez, F. , Sendra, E. , Lario, Y. , Fernandez‐Lopez, J. , Sayas‐Barbera, E. , & Perez‐Alvarez, J. (2006). Rheology of orange fiber enriched yogurt. Milchwissenschaft, 61, 55–59.

[fsn3470-bib-0009] Gorinstein, S. , Zachwieja, Z. , Folta, M. , Barton, H. , Pirtrowicz, J. , Zemser, M. , … Màrtín‐Belloso, O. (2001). Comparative contents of dietary fiber, total phenolics, and minerals in persimmons and apples. Journal of Agricultural and Food Chemistry, 49, 952–957. 11262055 10.1021/jf000947k

[fsn3470-bib-0010] Hashim, I. B. , Khalil, A. H. , & Afifi, H. S. (2009). Quality characteristics and consumer acceptance of yogurt fortified with date fiber. Journal of Dairy Science, 92, 5403–5407. 19841201 10.3168/jds.2009-2234

[fsn3470-bib-0011] Karimi, R. , Mortazavian, A. M. , & Amiri‐Rigi, A. (2012). Selective enumeration of probiotic microorganisms in cheese. Food Microbiology, 29, 1–9. 22029912 10.1016/j.fm.2011.08.008

[fsn3470-bib-0012] Krasaekoopt, W. , Bhandari, B. , & Deeth, H. (2004). The influence of coating materials on some properties of alginate beads and survivability of microencapsulated probiotic bacteria. International Dairy Journal, 14, 737–743.

[fsn3470-bib-0013] Lourens‐Hattingh, A. , & Viljoen, B.C . (2001). Yogurt as probiotic carrier food. International Dairy Journal, 11, 1–17.

[fsn3470-bib-0014] Makras, L. , Va Nacker, G. , & De Vuyst, L. (2005). Lactobacillus paracasei subsp. paracasei 8700:2 degrades inulin‐type fructans exhibiting different degrees of polymerization. Applied and Environment Microbiology, 71, 6531–6537. 10.1128/AEM.71.11.6531-6537.2005 PMC1287650 16269678

[fsn3470-bib-0015] Moreira, M. , Abraham, A. , & De Antoni, G. (2000). Technological properties of milks fermented with thermophilic lactic acid bacteria at suboptimal temperature. Journal of Dairy Science, 83, 395–400. 10750093 10.3168/jds.S0022-0302(00)74894-6

[fsn3470-bib-0016] Nagpal, R. , Kumar, A. , Kumar, M. , Behare, P. V. , Jain, S. , & Yadav, H. (2012). Probiotics, their health benefits and applications for developing healthier foods: A review. FEMS Microbiology Letters, 334, 1–15. 22568660 10.1111/j.1574-6968.2012.02593.x

[fsn3470-bib-0017] Pharmaceutiques, U. D. L. (1995). Dietary modulation of the human colonie microbiota: Introducing the concept of prebiotics. The Journal of Nutrition, 125, 1401–1412. 7782892 10.1093/jn/125.6.1401

[fsn3470-bib-0018] Saarela, M. , Virkajärvi, I. , Nohynek, L. , Vaari, A. , & Mättö, J. (2006). Fibres as carriers for Lactobacillus rhamnosus during freeze‐drying and storage in apple juice and chocolate‐coated breakfast cereals. International Journal of Food Microbiology, 112, 171–178. 16844253 10.1016/j.ijfoodmicro.2006.05.019

[fsn3470-bib-0019] Sendra, E. , Fayos, P. , Lario, Y. , Fernandez‐lopez, J. , Sayas‐barbera, E. , & Perez‐alvarez, J. A. (2008). Incorporation of citrus fibers in fermented milk containing probiotic bacteria. Food Microbiology, 25, 13–21. 17993372 10.1016/j.fm.2007.09.003

[fsn3470-bib-0020] Staffolo, M. D. , Bertola, N. , Martino, M. , & Bevilacqua, Y. A. (2004). Influence of dietary fiber addition on sensory and rheological properties of yogurt. International Dairy Journal, 14, 263–268.

[fsn3470-bib-0021] Thiago, L. R. L. D. S. , & Kellaway, R. C. (1982). Botanical composition and extent of lignification affecting digestibility of wheat and oat straw and paspalum hay. Animal Feed Science and Technology, 7, 71–81.

[fsn3470-bib-0022] Trachoo, N. , & Mistry, V. V. (1998). Application of ultrafiltered sweet buttermilk and sweet buttermilk powder in the manufacture of nonfat and low fat yogurts. Journal of Dairy Science, 81, 3163–3171.

[fsn3470-bib-0024] Zomorodi, S. , Aberoon, N. , & Khosrowshahi, A. . (2015). Increase the survival of Lactobacillus acidophilus and improved quality properties of senbiotic yogurt using apple and wheat fibers. Iranian Journal of Food Science and Technology, 12, 203–214.

